# Echocardiographic manifestations of mitochondrial disease with GTPBP3 gene mutations: A case report

**DOI:** 10.1097/MD.0000000000037847

**Published:** 2024-05-03

**Authors:** Qiaoli Tong, Yajing Miao, Hongning Yin

**Affiliations:** a Department of Cardiac Ultrasound, The Second Hospital of Hebei Medical University, Shijiazhuang, China.

**Keywords:** echocardiographic, mitochondrial disease, the GTPBP3 gene mutations

## Abstract

**Rationale:**

Mitochondrial diseases are a group of disorders in which mutations in mitochondrial DNA or nuclear DNA lead to dysfunctional oxidative phosphorylation of cells, with mutations in mitochondrial DNA being the most common cause of mitochondrial disease, and mutations in nuclear genes being rarely reported. The echocardiographic findings of mitochondrial diseases with nuclear gene mutations in children’s hearts are even rarer. Even more valuable is that we followed up the patient for 4 years and dynamically observed the cardiac echocardiographic manifestations of mitochondrial disease. Provide ideas for the clinical diagnosis and prognosis of mitochondrial diseases.

**Patient concerns:**

The patient was seen in the pediatric outpatient clinic for poor strength and mental retardation. echocardiography: mild left ventricular (LV) enlargement and LV wall thickening. Nuclear genetic testing: uanosine triphosphate binding protein 3 (GTPBP3) gene mutation. Diagnosis of mitochondrial disease.

**Diagnoses:**

Mitochondrial disease with GTPBP3 gene mutations.

**Outcomes:**

After receiving drug treatment, the patient exhibited a reduction in lactate levels, an enhanced physical condition compared to prior assessments, and demonstrated average intellectual development.

**Lessons subsections:**

For echocardiographic indications of LV wall thickening and LV enlargement, one needs to be alert to the possibility of hereditary cardiomyopathy, especially in children.

## 1. Introduction

Mitochondrial disease is a disease in which cells become dysfunctional due to DNA mutations, which can accumulate in multiple organs. This case introduces a pediatric patient with hypertrophic cardiomyopathy complicated with heart failure caused by mitochondrial nuclear gene mutations, and illustrates the clinical manifestations of mitochondrial disease from the child’s ultrasound and cranial magnetic resonance imaging (MRI). The child was followed up for 4 years, focusing on the progression of mitochondrial disease accumulation from the patient’s echocardiographic findings.

## 2. Case presentation

**Figure 1.**
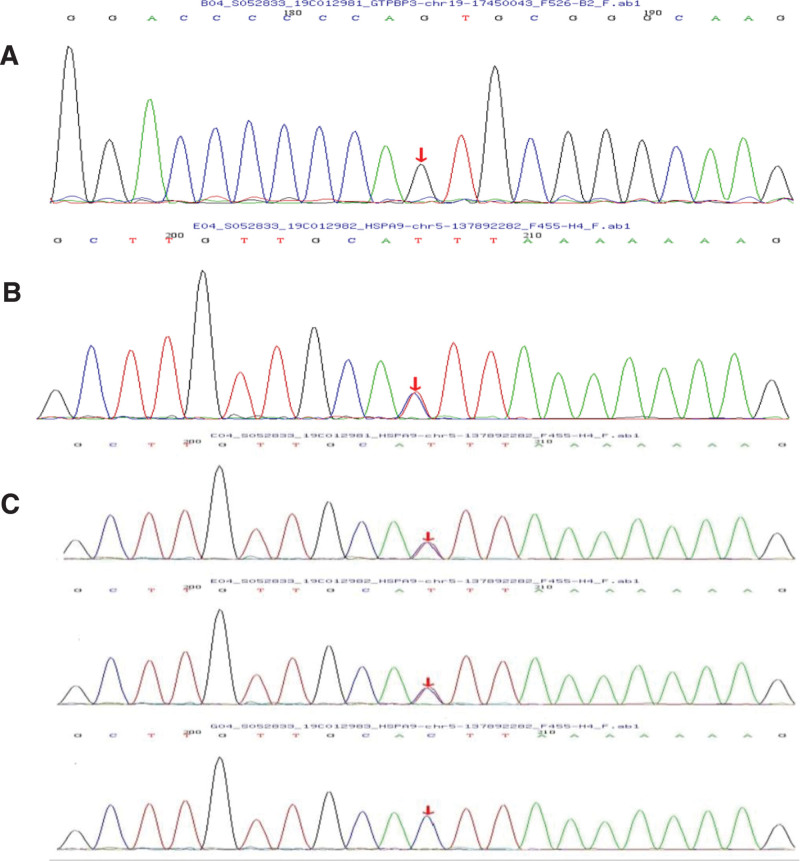
The findings of pedigree Sanger sequencing analysis were as follows. (A) The proband carried homozygous mutations on the GTPBP3 gene; (B) the father of the proband exhibited heterozygous mutations on the GTPBP3 gene; (C) the mother of the proband also had heterozygous mutations on the GTPBP3gene. GTPBP3 = guanosine triphosphate binding protein 3.

A 6-year-old girl was presented to the pediatric outpatient clinic in 2019 complaining of poor physical strength and mental retardation. On physical examinations, her temperature was 36.5°C, pulse rate was 94 beats/min, respiratory rate was 20 breaths/min, and blood pressure was 79/54 mm Hg. Her limb muscle strength was at grade-4 and the muscle tone was normal. Laboratory examinations indicated the lactate level of 4.8 mmol/L (reference range, 0.5–1.6 mmol/L), lactate dehydrogenase level of 273 U/L (reference range, 120–250 U/L), and creatine kinase level of 124 U/L (reference range, 40–200 U/L). Comprehensive urine organic acid analysis revealed elevated levels of octanedioic and azelaic acids, which might be correlated with the enhanced oxidative metabolism of fatty acids. Moreover, the amino acid and acylcarnitine profile analysis for genetic metabolic diseases indicated no significant abnormalities. According to the developmental examination for children aged 0 to 6 years, the child was experiencing mild developmental delays. Cranial MRI showed that there were multiple signal shadows of patchy long T2, high fluid-attenuated inversion recovery and high diffusion weighted imaging in bilateral basal ganglia, thalamus, mesencephalon, and bilateral cerebellar hemispheres. On echocardiography, the child exhibited mild left ventricular (LV) enlargement and LV wall thickening (interventricular septal thickness, 7.6 mm; LV posterior wall thickness, 7.6 mm; LV end-diastolic volume [LVEDV], 82 mL; LV ejection fraction [LVEF], 78%). Besides, no abnormalities were observed in the mitochondrial full-length genes of the child or her parents. The nuclear genetic test showed that the GTPBP3 gene of the child mutated and that her parents both carried heterozygous GTPBP3 variants (Fig. 1). (The relevant examination materials and data of the patient have been obtained with the consent of her guardian).

With the above findings, the child was diagnosed with mitochondrial disease. Accordingly, she was treated with drugs such as vitamin B1, coenzyme Q10, and L-carnitine. As her symptoms improved, she could take exercise of low-to-moderate intensity and her academic performance was below average. Then the child was regularly followed up in the pediatric outpatient clinic of our hospital between the ages of 6 and 10.

**Figure 2.**
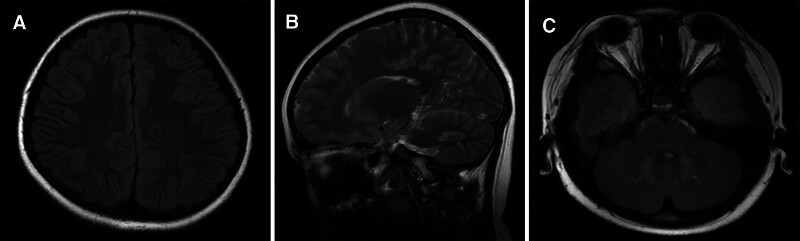
Cranial MRI showed that there were abnormal signs (White arrow) in right frontal. (A) and left parietal areas as well as symmetrical abnormal signals in bilateral cerebellum. (B and C) and corpora quadrigemina. MRI = magnetic resonance imaging.

**Figure 3.**
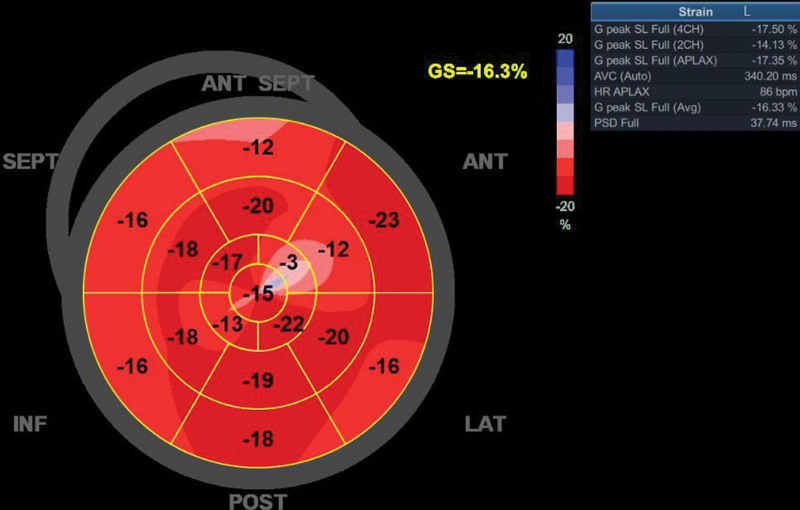
The echocardiography when the child was 10 revealed reduced LV-GLS. GLS = global longitudinal strain, LV = left ventricular.

This time, the child was reviewed in the pediatric outpatient clinic of our hospital with the chief complaint of limb muscle soreness. Through physical examinations, the information of her vital signs was normal. Additionally, she was undersized (height, 134 cm; weight, 34 kg) with grade-4 limb muscle strength and normal muscle tone. Her limb muscle strength declined. Laboratory examinations indicated that the levels of lactate and blood glucose were 5.0 and 6.0 mmol/L, respectively. Electrocardiogram revealed sinus rhythm and LV high voltages. On cranial MRI, there were abnormal signs in right frontal and left parietal areas as well as symmetrical abnormal signals in bilateral cerebellum and *corpora quadrigemina* (Fig. 2). Echocardiography illustrated that the child experienced LV wall thickening, LV enlargement, and reduced LV global longitudinal strain (GLS) (interventricular septal thickness, 10 mm; LV posterior wall, 10 mm; LVEDV, 103 mL; GLS, −16%; LVEF, 60%) (Video 1 and Fig. 3). In light of the above findings, the child was administered with zinc sulfate and captopril additionally. At a follow-up 2 months later, the soreness in her limbs had disappeared.

## 3. Discussion

In this case, the child visited our hospital due to poor physical strength and mental retardation. She was born at full term to non-consanguineous parents. Relevant examinations suggested that her lactate level was elevated, and cranial MRI showed that there were multiple signal shadows of patchy long T2, high fluid-attenuated inversion recovery and high diffusion weighted imaging in bilateral basal ganglia, thalamus, mesencephalon, and bilateral cerebellar hemispheres. Echocardiography indicated LV wall thickening and LV enlargement, implying that the child might suffer from inherited cardiomyopathy. After the genetic test, she was confirmed to have mitochondrial disease with GTPBP3 mutations. At a subsequent follow-up, echocardiography showed that her LV wall thickened from 7.6 to 10 mm and LVEDV increased from 82 to 103 mL. Besides, her LVEF was within the normal range while the LV-GLS reduced. Hence, the child was considered to have experienced an exacerbation of mitochondrial disease involving the myocardium.

Mitochondrial disease is manifested by the oxidative phosphorylation dysfunction in cells resulting from mitochondrial or nuclear DNA mutations. Mitochondrial DNA mutations are the most prevalent etiology of mitochondrial disease, while nuclear gene mutations are rarely reported as the cause of the disease. Mitochondrial disease involves multiple organ systems, especially organs with high energy demands such as the brain, skeletal muscle and heart. The manifestations of cardiac involvement include hypertrophic cardiomyopathy, dilated cardiomyopathy, arrhythmia and heart failure.^[1]^ It has been reported that 20% to 40% of the children with mitochondrial disease will suffer from cardiomyopathy,^[2,3]^ with hypertrophic cardiomyopathy being the most common disease type.^[4]^

The GTPBP3 gene, located on chromosome 19p13.11, is a nuclear-encoded gene responsible for the encoding of GTPBP3. Defects in GTPBP3 protein suppress the taurine modification of mitochondrial tRNA, which leads to mitochondrial translation defects, thereby triggering mitochondrial oxidative phosphorylation disorders and associated clinical syndromes, that is combined oxidative phosphorylation deficiency 23 (COXPD23).^[5]^ Typically, mitochondrial disease is inherited maternally. But COXPD23 is an autosomal recessive genetic disorder characterized by the early-onset hypertrophic cardiomyopathy, neurological symptoms, and hyperlactacidemia in childhood; the average age of onset is 1.7 years (3 months for children with homozygous mutations).^[6]^ So far, a total of 18 COXPD23 patients have been reported worldwide, and approximately **20** variants of the GTPBP3 gene have been identified. Based on clinical manifestations, Yan et al^[7]^ have classified the disease into severe and mild types. Severe COXPD23, which usually occurs during infancy, is characterized by rapidly deteriorating acute metabolic decompensation, with the specific manifestations of congestive heart failure, arrhythmia and serious hyperlactacidemia; the mild type, which typically occurs in early childhood, presents with cardiomyopathym, encephalopathy, mild hyperlactacidemia, and retarded development. Severe COXPD23 will finally lead to early death, while children with the mild type can survive to the age of 20 years. Research on genotype-phenotype correlations of COXPD23 patients noted^[6]^ that variant types and allelic status were substantially associated with the various types of COXPD23. It was also found that homozygous variants might be present in patients with severe COXPD23 (severe vs mild, 71.43% vs 18.18%), while those with mild type tended to carry compound heterozygotes. In the current case, the proband was discovered to have mild homozygous mutations. She carried c.872 A > G homozygous mutations and presented with mild developmental delay, encephalopathy, hyperlactacidemia and cardiomyopathy. Besides, the mitochondrial disease of the child involved the myocardium, and the characteristics of both hypertrophic and dilated cardiomyopathy were observed (LV wall thickened from 7.6–10 mm, and LVEDV increased from 82–103 mL). These conditions have not been reported by previous studies yet.

The diagnosis of mitochondrial disease is still challenging due to its heterogeneity. And in terms of its treatment, no literature is available at present. Symptomatic treatment is a commonly adopted method to combat the disease. Some researchers have recommended the classic treatment method of “mitochondrial cocktail,” which refers to the administration of coenzyme Q10, creatine, L-carnitine, vitamin B1, folic acid, and other antioxidants such as vitamin C and vitamin E. A previous investigation has confirmed that^[1]^ the application of antioxidants can partially alleviate patient’s clinical characteristics. For both symptomatic and asymptomatic patients with cardiac hypertrophy, angiotensin-converting enzyme inhibitors and beta-blockers are the recommended medications.^[8]^ In addition, although heart transplantation is controversial in the treatment of metabolic diseases, it has been successfully performed to treat patients with mitochondrial disease.^[9,10]^

Currently, only an extremely small number of COXPD23 patients have been identified worldwide. In this context, the present case study is able to update the information regarding the clinical diagnosis of COXPD23, and help expand the clinical phenotypes. We hope that a growing number of more intensive relevant studies will be conducted in the future so as to benefit COXPD23 patients.

## 4. Limitations

We were unable to acquire the 2019 cranial MRI results for the child diagnosed with mitochondrial disease, which hinders dynamic comparisons. Introducing three-dimensional measurements for LV volume in cardiac echocardiographic parameters would enhance precision. Including measurements for relative wall thickness and cardiac mass would further strengthen the analysis. These factors collectively contribute to the complexity of the diagnostic and treatment procedures. It is imperative that we maintain meticulous organization and preservation of all data for upcoming follow-ups.

## Acknowledgments

I would like to express my gratitude to all those who helped me during the conducting this case. Firstly, I gratefully acknowledge the help of Dr Yin, for his instructive advice and useful suggestions on my study. Secondly, I would like to thank Dr Miao for her writing assistance and image retention. Lastly, I am also deeply indebted to all the other members of the Department of Cardiac Ultrasound, Second Hospital of Hebei Medical University.

## Author contributions

**Supervision:** Hongning Yin.

**Validation:** Hongning Yin.

**Writing – original draft:** Qiaoli Tong.

**Writing – review & editing:** Yajing Miao.
